# Synergistic Reinforcing Effect of Hazelnut Shells and Hydrotalcite on Properties of Rigid Polyurethane Foam Composites

**DOI:** 10.3390/polym16212968

**Published:** 2024-10-23

**Authors:** Sylwia Makowska, Karolina Miedzińska, Agnė Kairytė, Krzysztof Strzelec

**Affiliations:** 1 Institute of Polymer and Dye Technology, Lodz University of Technology, 90-924 Lodz, Poland; karolina.miedzinska@dokt.p.lodz.pl (K.M.); krzysztof.strzelec@p.lodz.pl (K.S.); 2 Civil Engineering Research Centre, Vilnius Gediminas Technical University, Saulėtekio Av. 11, LT-10223 Vilnius, Lithuania; agne.kairyte@vilniustech.lt; 3 Laboratory of Thermal Insulating Materials and Acoustics, Faculty of Civil Engineering, Institute of Building Materials, Vilnius Gediminas Technical University, Linkmenu St. 28, LT-08217 Vilnius, Lithuania

**Keywords:** polyurethane, hazelnut shells, hydrotalcite, mechanical properties, flame-retardant properties

## Abstract

Recently, the development of composite materials from agricultural and forestry waste has become an attractive area of research. The use of bio-waste is beneficial for economic and environmental reasons, adapting it to cost effectiveness and environmental sustainability. In the presented study, the possibility of using hazelnut shell (HS) and hydrotalcite (HT) mineral filler was investigated. The effects of fillers in the amount of 10 wt.% on selected properties of polyurethane composites, such as rheological properties (dynamic viscosity, processing times), mechanical properties (compressive strength, flexural strength, hardness), insulating properties (thermal conductivity), and flame-retardant properties (e.g., ignition time, limiting oxygen index, peak heat release), were investigated. Polyurethane foams containing fillers have been shown to have better performance properties compared to unmodified polyurethane foams. For example, the addition of 10 wt% of hydrotalcite filler leads to PU composite foams with improved compression strength (improvement by ~20%), higher flexural strength (increase of ~38%), and comparable thermal conductivity (0.03055 W m^–1^ K^–1^ at 20 °C). Moreover, the incorporation of organic fillers has a positive effect on the fire resistance of PU materials. For example, the results from the cone calorimeter test showed that the incorporation of 10 wt% of hydrotalcite filler significantly reduced the peak of the heat release rate (pHRR) by ca. 30% compared with that of unmodified PU foam, and increased the value of the limiting oxygen index from 19.8% to 21.7%.

## 1. Introduction

Currently, PU materials are one of the most important polymers in terms of the share among polymer materials on a global scale, due to their versatility, the possibility of varying properties, and tailoring them to a specific application [1]. Thanks to their properties such as flexibility, durability, chemical resistance, and thermal insulation, polyurethanes are widely used in industries such as automotives, construction, furniture, coatings, refrigeration, adhesives, and many others. In 2022, the global polyurethane market was valued at USD 75.19 billion; additionally, as with the global polymer market, it is expected to grow at a compound annual growth rate of approximately 4.4% per year, by 2030 [2]. The most commonly used PU products are rigid and flexible foams, coatings, adhesives, and elastomers. On the other hand, the end-use industries most willing to use these materials are furniture, construction, electronics, automotive, footwear, and packaging industries [3,4,5].

An increased demand and production of polymeric materials, which are often treated as disposable, result in increased waste collection and negative environmental impact. Therefore, the polymer market has recently witnessed the optimal use of resources by recycling polymer materials to reduce waste and use of new raw materials, creating biodegradable polymers, and modifications involving the addition of natural additives to polymer materials to increase biocompatibility and reduce the negative impact on the environment [6,7,8,9].

Growing awareness of environmental issues and the need for sustainable manufacturing practices has led to the adoption of natural additives as one of the main avenues for the research and modification of polymer materials [10]. Polyurethanes are mainly based on petrochemical raw materials, which can have a detrimental impact on the environment over their entire life cycle. In opposition, additives derived from renewable materials offer a more environmentally friendly alternative [11,12,13,14,15,16,17,18]. It has been found that some of the natural additives can improve the mechanical properties of the composites to which they are added. They can improve tensile, compression, and flexural strength, and impact resistance. The use of natural fillers can also lead to a reduced shrinkage and distortion of the material, resulting in higher dimensional stability [19,20]. An additional advantage of natural additives is their relatively low price; sometimes even natural waste is used. By using natural materials as additives or replacements for synthetic ingredients, it is possible to achieve a reduction in production costs. This cost effectiveness can be particularly attractive in industries where cost competitiveness is a crucial factor, such as construction or insulation material production [21]. The use of natural additives is gaining importance due to their sustainability goals, cost effectiveness, improved mechanical properties, and alignment with consumer preferences. The path towards natural additives reflects a broader movement towards greener and more sustainable materials across industries, contributing to a more eco-friendly and responsible approach to polymer material production. Due to the variety and availability of natural materials, many have been reported in the literature as additives to polyurethanes. Studies have shown that their addition can improve the mechanical and application properties of composites. Natural modifiers may also contain reactive groups capable of reacting with isocyanates [19,20].

De Avila Delucis et al. [22] used forestry wastes as natural modifiers for polyurethane foams based on castor oil. They used wood, bark, cones, and needles from young pine trees; kraft lignin; and recycled paper sludge from industry wastes, in variable contents (1, 5, and 10 wt.%). The incorporation of forestry waste resulted in foams with more homogeneous cells. With the addition of wood, it was also possible to reduce the water absorption of composites. Their research showed that wood and kraft lignin presented better compatibility with the polyurethane system. In addition, these fillers enhanced the thermal and dimensional stability of composites [23]. Kakroodi et al. [23] investigated soy-based rigid polyurethane foams containing wood fiber. Their research showed that the addition of wood fibers increased the ratio of the tensile modulus to foam density, and reduced the density and compressive strength. Shear tests demonstrated that the use of a composite foam core significantly increased the maximum strength of wooden wall panels. The effect of paper waste sludge on the performance of polyurethane foams was also analyzed by Kairytė et al. [24]. They examined rigid polyurethane foams with the addition of various amounts (5–20%) of filler. The authors concluded that the rational amount of paper waste sludge particles is 5% by weight of the foam. They also showed that the addition of filler increased the density, compressive strength, and elastic modulus compared to neat polyurethane foam, as well as improved the thermal insulating capacity. Mosiewiecki et al. [25] used wood flour in castor oil-based rigid polyurethane foams. Due to the ability of wood flour to react with isocyanate, the authors found that foams with 15 wt.% filler were acceptable. These foams showed improved thermal stability but inferior mechanical properties, while the use of castor oil bio-polyol improved the fire behavior of the composites. Dukarska et al. [26] prepared rigid polyurethane foams filled with sawdust, a by-product of primary wood processing, as a filler in amounts of 5, 10, 15, and 20 wt.%. Their research showed that filler content over 10% resulted in significant structure defects. On the other hand, the addition of filler in the amount of 10% enabled a reduction in thermal conductivity and decreased the brittleness of the foam. The authors also observed an increase in water absorption and a decrease in mechanical properties such as flexural and compressive strength. However, these values were close to the parameters of commercially available materials. Oushabi et al. [27] modified polyurethane foams with the addition of 5, 10, and 20 wt.% of date palm particles. Their research showed that composite foams have comparative thermal insulation properties with unmodified foam. However, the mechanical and thermal properties of composites have been found to be competitive with commercially available insulation materials.

The field of natural fillers in polymer, especially polyurethane, applications is a diverse landscape in which each type of filler has its own set of distinctive properties and advantages. Some increase mechanical properties, and others contribute to sustainability and cost efficiency, while some fillers improve thermal stability, and others may have fire-resistant properties. As polyurethane materials develop in the future, realizing the full potential of these natural fillers will be essential. Their diverse properties and wide availability provide a promising avenue for the development of sustainable, high-performance materials in a variety of industries.

Despite the use of many bio-fillers, new lignocellulosic fillers are still being investigated, which would be able to successfully improve the properties of the insulating materials obtained and, in addition, would be a waste product for which a sustainable disposal option could be found. An example of a filler that meets the requirements is hazelnut shells. Hazelnut is one of the most popular nuts around the world. The most commercially cultivate species of hazelnut trees is the European hazel (*Corylus avellana* L.) [28]. The global production of in-shell hazelnuts has been on the rise recently, exceeding 1 million metric tons in 2020 [29]. Interestingly, the consumption of fresh nuts accounts for only about 10% of their share, while the vast majority are diverted to further industrial processes. Before further processing, the shelled nuts are split, leaving significant amounts of the by-product shells, which make up as much as 50–55% of their total weight, resulting in a substantial amount taking into account global production [30]. As a waste material, hazelnut shells are used mainly as fuel for heating [31,32]. Hazelnut shells are a lignocellulosic material, meaning that they consist mainly of lignin at 40–50%, cellulose at 17–29%, and hemicellulose at 13–29% [33]. Lower contents of protein, fat, and ash are also reported. The exact composition of lignocellulosic materials will vary depending on the species, the geographical location of the crop, and related conditions. Hazelnut shells are widely available, inexpensive, and environmentally friendly, and their addition as a filler can improve the mechanical and processing properties of composites [34,35].

Given that the main drawback of PU foams is their high flammability, this study focuses on the physical functionalization of hazelnut shell hydrotalcite, a flame-retardant compound, using a high-energy ball milling process. Given the favorable properties of hazelnut shells and hydrotalcite, it is expected that the PUR composites developed in this study will exhibit exceptional mechanical and thermal properties, expanding their application in construction. Accordingly, the effect of the developed bio-fillers on the mechanical, thermal, insulation, and performance properties of PUR composites will be investigated.

## 2. Materials and Methods

### 2.1. Materials

Stapanpol PS-2352 (polyester polyol) was purchased from Stepan Company (Northfield, IL USA); Purocyn B (4,4′-diphenyl-methane-diisocyanate) was provided by Purinova (Bydgoszcz, Poland); Tegostab B8513 (silicone surfactant), Kosmos 75 (Potassium octoate), Kosmos 33 (Potassium acetate), pentane (physical blowing agent), and cyclopentane (physical blowing agent) were purchased from Evonik (Essen, Germany); hydrotalcite (Mg_6_Al_2_(CO_3_)(OH)_16_ 4H_2_O) was provided by Sigma-Aldrich (Saint Louis, MO, USA); and hazelnut shells were supplied by a local company (Lodz, Poland).

### 2.2. Methodology

#### 2.2.1. Filler Preparation

The grinding was undertaken in 5 two-minute series with the maximum speed of rotation using the Monsieur Cuisine SKMC 1200 E5 (Lidl Stiftung & Co. KG, Neckarsulm, Germany) equipped with cutting knives, with rotation set to the right. After grinding, the resulting fillers were dried in an oven at 30 °C for 2 days to evaporate any moisture. To optimize and facilitate the process, the fillers were not sieved or fractionated after grinding.

#### 2.2.2. Particle Size of Fillers

The fillers’ particle size was determined using a Zetasizer NANOS90 instrument (Malvern Panalytical, Westborough, MA, USA). The measurement is based on the dynamic light scattering phenomenon and the analysis of the Brownian motion of the particles present in the examined sample. The mass concentration of the fillers’ particles in the polyol was 0.04 g/L.

#### 2.2.3. Optical Microscopy

The surface of the fillers and morphology of the polyurethane foams were studied using a LAB 40 M optical microscope from OPTA-TECH (Warsaw, Poland). For a more detailed analysis, images were taken at different magnifications (50× and 100×). An image analysis was carried out using graphical computer software. The anisotropy coefficient was identified as the cell length-to-width ratio. The parameters represented in the tables were determined from at least a few microscope images and the images presented in the experimental section are chosen overview images.

#### 2.2.4. Fourier Transform Infrared Spectroscopy

The presented materials were examined using Fourier transform infrared spectroscopy (FTIR), which enables the analysis of functional groups present in the samples. The measurement was performed using a Thermo Scientific Nicolet 6700 spectrometer (Waltham, MA, USA) equipped with a Smart Orbit ATR diamond tool. The spectra were recorded in the range of 4000–400 cm^−1^ (number of scans—128, resolution—4 cm^−1^). All spectra were recorded with respect to background spectra. The obtained spectra were analyzed using OMNIC 9.2.86 software (Waltham, MA, USA).

#### 2.2.5. Dynamic Viscosity

The polyol systems’ dynamic viscosity was evaluated with a Brookfield DV-II+ viscometer (Middleborough, MA, USA), following the ISO 2555 standard [36]. Before taking measurements, the device was calibrated using a special adjustment ring. The liquid to be analyzed, approximately 1 cm^3^ in volume, was placed in a sample container, which was then assembled in the apparatus and locked with a special clamping handle. Viscosity measurements were conducted at 23 °C at the following shear rates: 10, 20, 50, and 100 rotations per min.

#### 2.2.6. Apparent Density

The apparent density of polyurethane foams was determined as the weight-to-volume ratio of the sample, according to ISO 845 [37]. The samples were measured to hundredths of a millimeter and weighed to hundredths of a gram, and then their density was calculated. Measurements were taken on 40 mm × 40 mm × 40 mm specimens at room temperature.

#### 2.2.7. Mechanical Properties

The hardness of the polyurethane foams was established on samples measuring 100 mm × 10 mm × 5 mm, using a Zwick/Roell Type 00 Shore hardness tester type 00 by Zwick/Roell (Zwick/Roell Group, Ulm, Germany) equipped with a 1.2 mm diameter ball indenter. During the test, at least ten measurements were carried out for each sample, consisting of the indenter being driven into the material, and the results are displayed as an average.

The compressive strength of polyurethane foams was determined in accordance with ISO 844 [38,39]. The measurement was carried out on 80 mm × 80 mm × 50 mm specimens, which were subjected to compression using a Zwick Z100 testing machine (Zwick/Roell Group, Ulm, Germany) with a 2 kN load cell. Compressive strength was measured in the parallel direction to the foam growth, as the average of at least four samples of the load ratio, causing a 10% deformation of the cross-section of the samples.

The flexural strength of the polyurethane foams was determined in terms of ISO 178 [39]. The test was carried out on 100 mm × 10 mm × 5 mm specimens, which were bent at 2 mm min^−1^ using a Zwick Z100 testing machine (Zwick/Roell Group, Ulm, Germany). At least five measurements were taken for each specimen, and the results are presented as an average.

#### 2.2.8. Water-Related Properties

The water absorption of polyurethane foams was measured in accordance with ISO 2896 [40], and the test was conducted on 40 mm × 40 mm × 40 mm specimens. Before immersing in water, the samples were dried and weighed. Then, they were immersed in distilled water to a 10 mm depth. Once the samples were removed from the water after 24 h, they were dried with dry filter paper and then weighed. The measurement was taken at least four times for each sample, with the result displayed as an average.

A study of the contact angle by the sessile drop method was also carried out. The test was performed on 100 mm × 10 mm × 5 mm samples, employing OCA 15EC goniometer from DataPhysics Instruments GmbH (Filderstadt, Germany) The measurement consisted of applying a 1 μL drop of water to the sample surface cut from the inside of the foam and gauging the angle at the point of contact between the three phases: solid, liquid, and gas. The values of the contact angle were gauged at least fifteen times for each sample, and the result was displayed as an average.

#### 2.2.9. Thermogravimetric Analysis

The thermal stability of the polyurethane foams was evaluated with a Mettler Toledo TGA/DSC1 thermogravimetric analyzer (Mettler Toledo, Greinfensee, Switzerland). The analysis consisted of a study of mass change as a function of temperature. Thermal decomposition was performed in the 25 °C to 800 °C temperature range (heating rate of 10 °C min^−1^) and in an inert gas atmosphere (flow rate of 50 mL min^−1^). The temperatures of the thermal decomposition stages were established based on the inflection points on the DTG chart.

#### 2.2.10. Burning Behavior

The combustion behavior of polyurethane foams was assessed by the ISO 5660 standard [41]. During the investigation, the following combustion properties were evaluated: the ignition time (IT), total heat release (THR), total smoke release (TSR), carbon monoxide yield (COY), carbon dioxide yield (CO_2_Y), and maximum average heat release rate (MARHE). The investigations were performed on 100 mm × 100 mm × 50 mm samples using a cone calorimeter from S.Z.T.K. TAPS (Maciej Kowalski Company, Lodz, Poland). Each sample was foiled with aluminum foil and burned at a 35 kW m^−2^ external heat flux.

#### 2.2.11. Thermal Conductivity

The thermal conductivity of polyurethane foams was evaluated in terms of ISO 8301 [42], employing the HFM 446 Lambda Series (NETZSCH, Selb, Germany). The study was conducted on 200 × 200 × 20 mm^3^ specimens at average temperatures of 10, 20, and 40 °C under a load of 2 kPa. The temperature presented in the analysis was an intermediate value, while the temperatures of the plates on the two sides of the analyzed foam differed by 11 °C at each stage and were 4.5, 14.5, and 34.5 °C for the lower plate and 15.5, 25.5, and 45.5 °C for the upper plate.

### 2.3. Synthesis of Polyurethane Foams

Synthesis of all polyurethane foams was carried out via a ‘one-shot’ method. The prepared amounts of polyol and auxiliaries were inserted into a container and mixed together using a mechanical stirrer at a speed of 1000 rpm for 30 s to obtain component A. The isocyanate (component B) was subsequently added to the container and the components were mixed at 1000 rpm for 30 s until uniformly combined. The polyol and isocyanate were blended in a ratio of 1:1.6 according to the supplier, in order to ensure a complete reaction. The mixture was left in the container, allowing for a free vertical growth. For the addition of single hazelnut shells or hydrotalcite, 10 parts were used relative to the polyol. Meanwhile, for the fillers’ combinations, their mutual ratios (3:1, 1:1, and 1:3) were used, which together also amounted to 10 parts by weight relative to the polyol. The compositions of polyurethane foams are shown in Table 1.

To better evaluate the characteristics of foam growth, maximum temperature and characteristic synthesis times were measured during synthesis; start time was measured from mixing the ingredients to the start of foam growth; expansion time was measured from the start of growth to when the maximum foam height was reached; and stabilization time was measured from reaching maximum height to when the foam surface lost its tack. Characteristic synthesis times were measured using a stopwatch, based on an optical analysis of the transition between consecutive stages of foam synthesis. The maximum synthesis temperatures were measured via a thermocouple and noted. After synthesis, the polyurethane foams were conditioned for at least 48 h under room temperature and then cut into samples for testing.

## 3. Results and Discussion

### 3.1. Fillers’ Characterization

**Figure 1:**
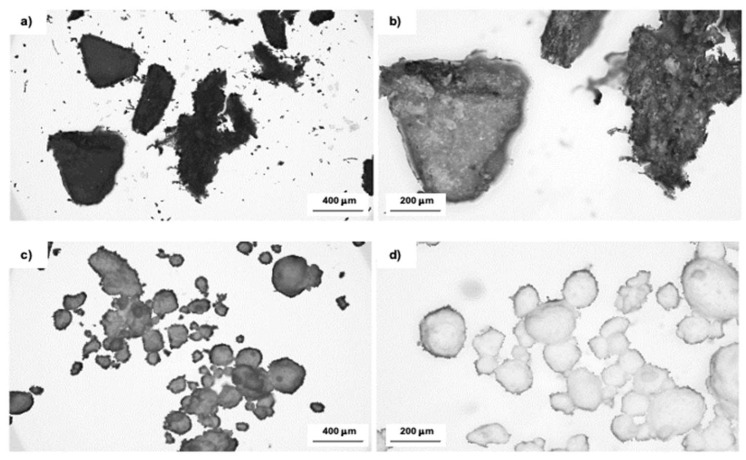
Optical images of hazelnut shells (**a**,**b**) and hydrotalcite (**c**,**d**) at magnifications of 50 (**a**,**c**) and 100 (**b**,**d**).

The surface structure of the hazelnut shell filler and the mineral filler was investigated using optical microscopy techniques. Figure 1a,b show images of hazelnut shells obtained at 50× and 100× magnifications. Based on these images, it can be noticed that hazelnut shells are heterogeneous in shape and have rougher surfaces, which may be due to the natural structure of the shells or the presence of remnants of membranes surrounding the nuts, fused to the shells. A waxy layer can be observed on their surface. According to Figure 1c,d, hydrotalcite occurred in the form of spherical particles that agglomerated when exposed to moisture, and showed similar behavior in polyol when unmixed. These particles showed a spherical shape with a kind of hairy surface.

**Figure 2:**
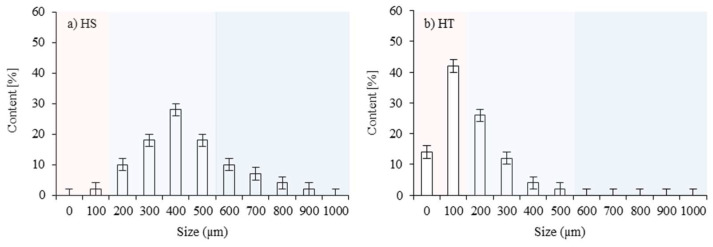
Size distribution of fillers: (**a**) hazelnut shells and (**b**) hydrotalcite.

According to the results of the dynamic light scattering (DLS) method, presented in Figure 2a, the hazelnut shells showed a wide (100–1000 µm) and uniform particle size range. The largest share of 64% occurred in the 300–500 µm range with a peak of 28% at ~400 µm. According to Figure 2b, hydrotalcite particles did not exceed 600 µm while the largest 42% share was observed for particles having ~100 µm.

**Figure 3:**
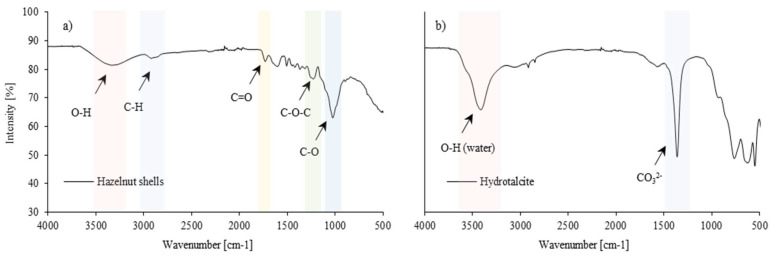
FTIR spectra of (**a**) hazelnut shells, and (**b**) hydrotalcite.

The chemical structure of the fillers, hazelnut shells, and hydrotalcite was investigated by the FT-IR technique. The spectrum of the hazelnut shells is shown in Figure 3a. The bands obtained on the FT-IR spectrum can be assigned to the following functional groups: the 3300 cm^−1^ band is the stretching vibration of the hydroxyl group O-H, the 2990–2800 cm^−1^ band is the stretching vibration of the C-H group, and the peaks located at wavelengths of 1730 cm^−1^ by 1600 cm^−1^ correspond to the stretching vibration of the carbonyl group C=O [43,44,45]. The peaks at 1230 cm^−1^ and 1030 cm^−1^ correspond to stretching vibrations of the C-O-C and C-O groups, respectively [46,47,48]. The peaks at 1230 cm^−1^ and 1030 cm^−1^ correspond to stretching vibrations of the C-O-C and C-O groups, respectively [36,37,38]. A broad band in the wave number range of 3500–3000 cm^−1^ may be related to C–C and C–H stretching [46,47,48].

Considering the hydrotalcite FTIR spectrum (Figure 3b), the broad band in the range of 3700–3200 cm^–1^ with a peak at 3410 cm^–1^ is assigned to the O-H stretching vibration of water molecules. The weak peaks at 2915 cm^–1^ and 2850 cm^–1^ are attributed to the stretching vibrations of C–H bonds, which may be related to organic impurities on the hydrotalcite surface. The band at 1365 cm^–1^ indicates the presence of carbonate groups and is related to the antisymmetric stretching vibration of carbonate ions in the interlayer. The bands at 765, 630, 550, and 440 cm^–1^ might be related to metal–oxygen stretching, and metal–hydroxyl bending vibrations within the hydrotalcite structure, as at similar wavenumbers in synthetic Mg/Zn/Al-hydrotalcites, they were ascribed to Mg-OH and Al-OH translation modes [49,50,51].

**Figure 4:**
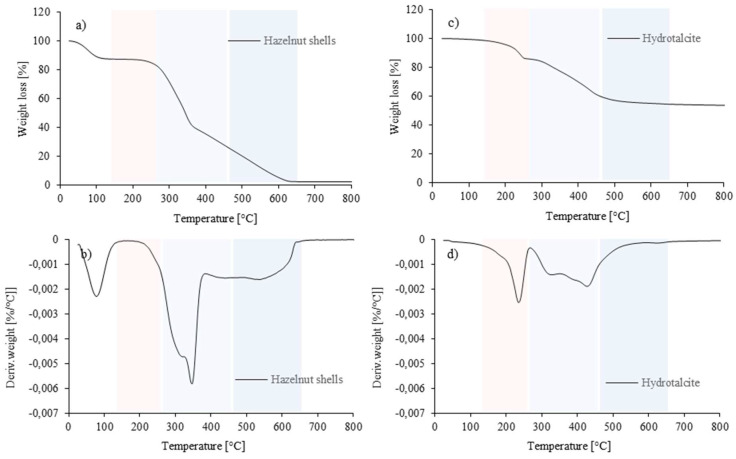
Thermogravimetric and derivative thermogravimetric results of (**a**,**b**) hazelnut shells, and (**c**,**d**) hydrotalcite.

A thermogravimetric analysis is important for several reasons. First, it makes it possible to analyze changes in the mass of a material as a function of temperature, which makes it possible to determine decomposition temperatures. To determine the thermal stability of hazelnut shells and hydrotalcite, a thermogravimetric and a derived thermogravimetric analysis were performed. Based on the inflection points on the DTG curve, the decomposition stages of the fillers and char residue at 600 °C were determined. The results are presented in Figure 4, and summarized in Table 2.

Hazelnut shells, which belong to the family of lignocellulosic materials, consist mainly of cellulose, hemicellulose, and lignin. According to literature data, the thermal decomposition of cellulose, hemicellulose, and lignin occur in the temperature range 310–400 °C, 210–235 °C, and 160–900 °C, respectively [45,52]. According to the data presented in Table 2 and Figure 4a,b, the second stage of thermal decomposition (associated with the thermal decomposition of cellulose and hemicellulose) occurred at 347 °C and 319 °C. The third thermal decomposition stage associated with lignin degradation occurred at 534 °C. Char residue at 600 °C was 5.7%. The thermogravimetric characteristics of hydrotalcite are summarized in Table 2 and presented in Figure 4c,d. In this case, on the DTG plot, there are two lost areas observed, the first up to 270 °C and the second in the 300–600 °C range. The first area with a peat at 235 °C is associated with the evaporation of physical and interlayer water. The second stage, with the main peak at 426 °C and a shoulder at 328 °C, according to the literature, may be related to the dihydroxylation and decomposition of carbonate anions in the hydrotalcite interlayer space [53,54,55]. Hydrotalcite showed residual content at 53.4%, indicating relatively low thermal stability.

### 3.2. Properties of Foams with Hazelnut Shells and Hydrotalcite

**Figure 5:**
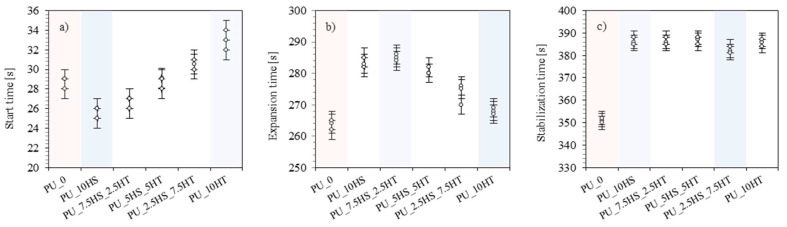
Characteristic times of PU foams filled with hazelnut shells and hydrotalcite—(**a**)—start time, (**b**)—expansion time, (**c**)—stabilization time.

When analyzing the characteristic times of foams with combinations of hazelnut shells and mineral fillers, it can be observed that foams with fillers generally showed extended synthesis times compared to the reference foam (Figure 5). All the foams showed similar start times ranging from 26 to 32 s. For foams with hydrotalcite addition, both expansion and stabilization times show a tendency to reach lower values with increasing hydrotalcite content. The values of expansion times were 285, 282, and 276 s for PU_7.5HS_2.5HT, PU_5HS_5HT, and PU_2.5HS_7.5 HT foams, respectively, while PU_10HS and PU_10HT foams reached 285 and 268 s. The values of stabilization times of foams with the addition of filler combinations were ideally between 384 s (time corresponding to PU_10HT) and 388 s (time corresponding to PU_10HS) and reached 386, 385, and 384 s for PU_7.5HS_2.5HT, PU_5HS_5HT, and PU_2.5HS_7.5HT, respectively. The total time of the synthesis was 699 s for the PU_10HS; 698, 696, and 691 s for foams with hazelnut shells/hydrotalcite fillers; and 684 s for the PU_10HT foam. It can be seen that since the times of the synthesis steps decrease with increasing hydrotalcite content, the total times of the process also follow this trend, decreasing towards the PU_10HT foam. A similar relationship was shown in previous work that concerned the modification of rigid PU foams with other types of organic and inorganic fillers [56,57,58].

The results of maximum temperature of the synthesis of polyurethane foams with the addition of hazelnut shells and hydrotalcite are presented in Table 3. In terms of the maximum temperature of the process, the effect of the fillers was observed to increase the temperature compared to the reference foam, which reached 121.8 °C. The trend of higher temperatures with higher mineral filler content was maintained for foams containing hazelnut shells/hydrotalcite combinations. However, in this case, the PU_7.5HS_2.5HT foam exhibited a temperature of 126.7 °C, which was slightly lower than that of the PU_10HS foam containing hazelnut shells alone (127.1 °C). The other foams in the series reached 128.7 °C (PU_5HS_5HT foam) and 128.9 °C (PU_2.5HS_7.5HT foam), while PU_10HT foam reached 132.5 °C.

**Figure 6:**
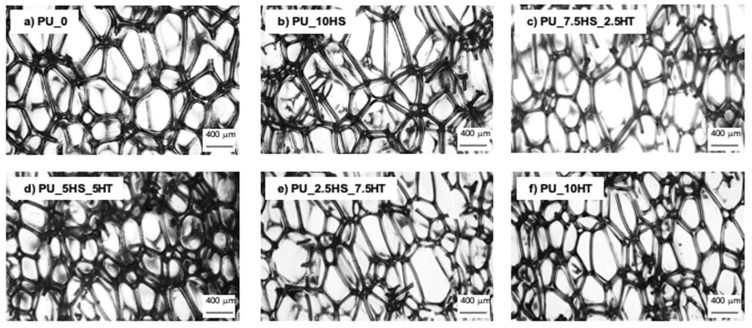
Morphology of polyurethane foams (**a**), polyurethane foams with hazelnut shells (**b**), polyurethane foams with the addition of hazelnut shells and hydrotalcite (**c**–**e**), and polyurethane foams with the addition of hydrotalcite (**f**).

The cellular structure distinguishes foams from other polyurethanes, but also determines many important properties that define their subsequent use. The cell structure affects the density of the foams and therefore the mechanical properties as well as the thermal and acoustic insulation capabilities [59,60]. Microscopic images of polyurethane foams and polyurethane composites filled with the addition of hazelnut shells and hydrotalcite are shown in Figure 6. Structure and rheological parameters are summarized in Table 4.

From the images, it can be seen that the foams exhibit a typical polyhedral structure of cells, separated by ribs. The cells undergo noticeable elongation in the direction of foam growth, which is the direction of least resistance to the expansion of the foaming gas. Interestingly, despite the increased viscosity of the polyol system, the PU_10HS foam exhibited a much lower density (32.51 kg m^–3^) and larger average cell diameter (528 µm) than the reference foam. This may be due to the high moisture content in hazelnut shells. This suspicion was raised in the case of the TGA analysis with a marked loss of weight in the first stage of thermal decomposition. During the foam growth stage, the moisture contained in the filler may have evaporated, acting as an additional foaming agent, leading to an increase in cell size and lowering the apparent density. It can also be noted that the anisotropy for the PU_10HS foam has a lower value when compared to the reference foam. This may indicate that the bubbles growing in the direction of foam growth may have simultaneously merged in other directions under the influence of the porophore, reducing the anisotropy coefficient.

The cellular structure of foams filled with hazelnut shells/hydrotalcite is presented in Figure 6c–e. The foams show a typical polyhedral arrangement of cells separated by interconnecting ribs and, as in the previous cases, the foams show cell elongation in the direction of growth, which is described by the anisotropy coefficient. Based on this parameter, it can be observed that the foams with the addition of hazelnut shells/hydrotalcite combinations achieved anisotropy coefficient values of 1.70–1.71, slightly higher than the 1.69 achieved by the PU_10HT foam. In general, all foams with hazelnut shells and mineral fillers showed a higher anisotropy coefficient compared to the reference foam (1.62), with a maximum difference of about 6%. The analysis of the data in Table 4 shows that all polyol systems with the addition of fillers (both single fillers and combinations) had significantly increased dynamic viscosity at 10 RPM compared to the reference foam. Generally, the polyol systems containing combinations of hazelnut shells with hydrotalcite showed intermediate viscosity values between the extremes reached by systems containing single fillers, with an increasing or decreasing trend between them. Hazelnut shells/hydrotalcite foams exhibit an almost perfect trend of increasing dynamic viscosity (from 2490 mPa × s for PU_10HS to 2670 mPa × s for PU_2.5HS_7.5HT), increasing apparent density (from 32.51 kg m^–3^ for PU_10HS, through 33.17–35.91 kg m^–3^ for hazelnut shells/hydrotalcite foams, up to 40.45 kg m^–3^ for PU_10HT), and decreasing average cell size (from 528 to 439 µm) with increasing hydrotalcite content.

From the presented data, it can be noticed that the addition of hazelnut shell fillers increased the dynamic viscosity of the polyol systems, reduced the average cell size, and increased the apparent density compared to the reference foam. The foams with the addition of hazelnut shells/hydrotalcite combinations also exhibited increased cell anisotropy. Overall, both the dynamic viscosity of polyol systems and the apparent density of polyurethane foams increased, and the average cell size decreased with increasing mineral filler content. A similar relationship was demonstrated in the work of Bonab [61] and Bartczak [62], who showed that the lower the filler content, the more uniform the structure of the foam composites obtained.

**Figure 7:**
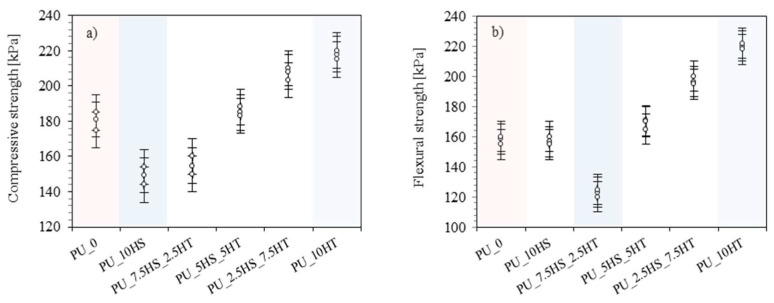
Effect of hazelnut shells and hydrotalcite fillers on (**a**) compressive strength and (**b**) flexural strength of polyurethane foams.

The mechanical properties of foams are an important aspect in the context of their use in construction [59,60]. Depending on the application, foams should retain their structure and not deform under load. To evaluate the effect of the combination of natural and mineral fillers on the mechanical properties of polyurethane foams, hardness, compressive strength, and flexural strength tests were conducted, the results of which are shown in Table 4 and Figure 7a,b.

Analyzing the effect of the addition of combinations of hazelnut shells and mineral fillers, it can be noticed that all the foams with the addition of fillers showed better results in terms of hardness than the reference foam. Here, it can be observed that foams with a combination of fillers achieved intermediate results between PU_10HS foam (62.8 °Sh) and the highest with the individual mineral filler—hydrotalcite (67.5 °Sh). In general, you can see a trend that the higher the density of the foam, the higher the hardness. The outstanding foam is the PU_2.5HS_7.5HT, which achieves a result of 67.3 °Sh, which is more similar to the PU_10HT foam than to the other combinations with hazelnut shells.

When analyzing the effect of the addition of a combination of hazelnut shells and mineral fillers, it can be observed that all foams with the addition of fillers showed higher compressive strength results compared to the PU_10HS foam (compressive strength of 149 kPa, and flexural strength of 157 kPa), which showed a significantly reduced apparent density compared to the reference foam and thus also interior mechanical properties. From the results obtained, it can be observed that for all types of mineral fillers, for both compressive and flexural strength, there is a trend that as the content of mineral fillers increases, the mechanical properties achieve higher results. In the case of compressive strength, the trend is practically perfect, and the lowest result is observed for the foam containing only hazelnut shells, while the highest results are obtained for the foams containing only mineral fillers, in line with the aforementioned trend. For flexural strength, there is also a trend toward higher results with increasing mineral filler content, but foams containing combinations of 7.5 parts by weight of hazelnut shells and 2.5 of all mineral fillers achieve even lower results than PU_10HS foam. For foams with the addition of combinations of hazelnut shells and mineral fillers, it can be concluded that the greatest influence on the mechanical properties of the foams is their apparent density, which reached higher values as the content of mineral fillers increased. Similar results were also obtained in previous works concerning the modification of rigid PU foams with the addition of organic and inorganic fillers [63,64,65,66]. For example, Liszkowska et al. [67] showed that PU foams modified with the addition of cinnamon extracts were characterized by better mechanical properties, due to the presence of polyphenols, which, reacting with isocyanate groups, increased the crosslinking of PU composites.

**Figure 8:**
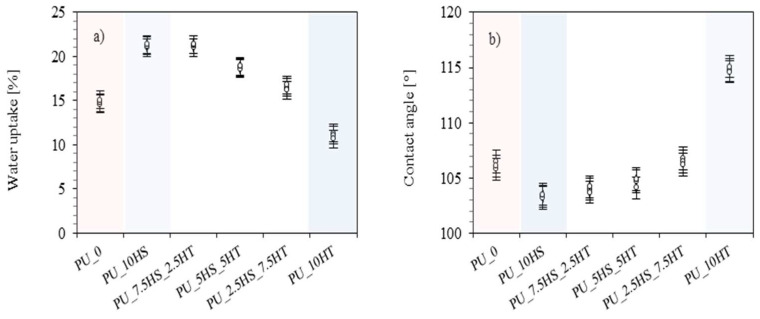
Results of (**a**) water uptake and (**b**) contact angle of PU foams.

The results of the water uptake and contact angle of PU foams are presented in Figure 8a,b. When analyzing the results of polyurethane foams with the addition of hazelnut shells, the following trend can be noticed—as the content of hydrotalcite increases and the content of hazelnut shells decreases, water absorption decreases. In this case, the tendency applies to all foams containing hazelnut shells mixed with hydrotalcite, which is also reflected in the results of the contact angle, the values of which increase as the content of hydrotalcite increases. The water absorption results of all foams containing hazelnut shells in combination with mineral fillers achieved intermediate results between the extreme water uptake values of PU_10HS (21.0%) and the foams with hydrotalcite filler—PU_10HT (11.4%). The water absorption of the polyurethane foams with the addition of hazelnut shells was as follows: with an increase in the content of hydrotalcite, the foams reached a water absorption of 21.0, 18.8, and 16.5%, respectively. Thus, all foams containing hazelnut shells combined with hydrotalcite achieved higher water absorption than the reference foam (14.6%). The dependence of the water absorption can be found in the density of the foams. This density, which was relatively low for the foam with hazelnut shells alone (high water absorption), increased with increasing mineral filler content until it reached maximum densities for foams without hazelnut shells (low water absorption).

**Figure 9:**
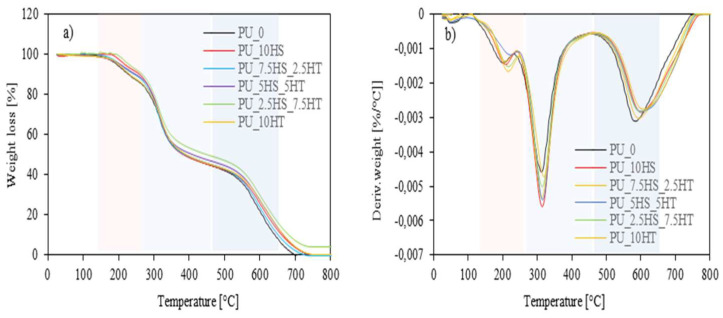
(**a**) Thermogravimetric (TGA) and (**b**) derivative thermogravimetry (DTG) results of polyurethane foams with the addition of hazelnut shells and hydrotalcite.

A thermogravimetric analysis provides the ability to study the degradation of polyurethane foams at different temperatures, which is essential for predicting their service life in the desired application. To investigate the influence of mineral fillers on the thermal stability of polyurethane foams, a thermogravimetric analysis and derived thermogravimetric analysis were conducted, the chart of which is shown in Figure 9a,b, and the results are summarized in Table 5. Analyzing the results of polyurethane foams with the addition of combinations of hazelnut shells and mineral fillers, it can be observed that, in general, the addition of all fillers increased the temperatures of subsequent thermal decomposition stages and the char residue at 600 °C compared to the reference foam. For foams with hydrotalcite added, PU_7.5HS_2.5HT foam reaches a temperature of 207 °C, also corresponding to PU_10HT foam, while PU_2.5HS_7.5HT and PU_5HS_5HT foams reach 216 and 221 °C, respectively, which are higher compared to the temperature corresponding to PU_10HT foam (212 °C). The second stage for all hazelnut shells/hydrotalcite foams was observed at 314 °C, which is also the temperature of PU_10HS and PU_10HT foams. The third stage for foams with combinations of hazelnut shells and hydrotalcite was observed at 599 °C (also corresponding to PU_10HS foam) for PU_5HS_5HT and PU_2.5HS_7.5HT foams and at 594 °C for PU_7.5HS_2.5HT. When analyzing the char residue content at 600 °C, a trend can be observed that the percentage of carbonization increases with increasing hydrotalcite content. However, foams with mixed fillers exceed the results of foams with single fillers, 25.9% (PU_10HS) and 26.3% (PU_10HT), reaching 24.0% for PU_7.5HS_2.5HT foam, 27.3% for PU_5HS_5HT foam, and even 30.1% for PU_2.5HS_7.5HT foam. The char content of the foam with hydrotalcite addition was 26.3%.

When analyzing the foams with hazelnut shells and hydrotalcite, it can be observed that all foams performed better than the reference foam, both in terms of higher temperatures of subsequent decomposition stages and higher char content at 600 °C. For foams with the addition of hydrotalcite, the char content at 600 °C increased with increasing mineral filler content. The obtained results imply that hazelnut shells and hydrotalcite fillers can improve the thermal stability of polyurethane foams by raising the temperatures of the following stages of decomposition and increasing the residual carbon content at 600 °C. The incorporation of mineral fillers such as hydrotalcite can act as a thermal barrier. These inorganic fillers can reflect, absorb, and dissipate heat, delaying the thermal degradation of the polyurethane matrix, which can result in higher decomposition temperatures [68,69]. In addition, it can be concluded that filler particles can act as crosslinking points between PU chains, reducing heat transfer through the composite structure. The higher crosslinked structure of PU composites effectively reduces the amount of volatile compounds that are released during the thermal degradation process. A similar relationship was also found in previous works as well [70,71,72].

**Figure 10:**
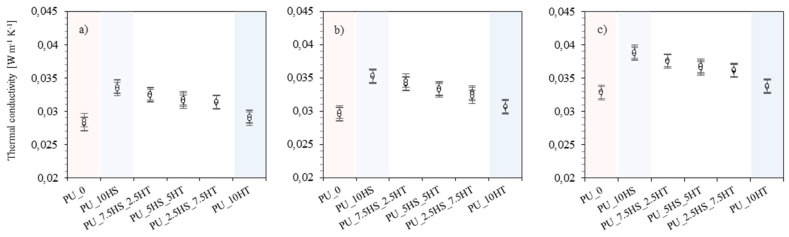
Thermal conductivity of foams at average temperatures of (**a**) 10, (**b**) 20, and (**c**) 40 °C.

Thermal conductivity (W m^–1^ K^–1^) is one of the key parameters that determine the thermal properties of materials [73]. It provides a measure of a material’s ability to conduct heat, which is particularly important in the case of thermal insulation materials used to minimize heat transfer between two areas of different temperature. For thermal insulation materials, lower l values are desirable, indicating lower thermal conductivity, which means a better thermal insulation of the material. To determine the effect of natural fillers on this key parameter of polyurethane foams, a thermal conductivity analysis was performed at three average temperatures of 10, 20, and 40 °C, with equal temperature differences between the upper and lower plates, and the results are shown in Figure 10a–c.

In general, thermal conductivity increased as the average analysis temperature increased. This is likely due to increased motion and kinetic energy of molecules in the material, increasing its ability to conduct heat. Furthermore, it can be noticed that, at all three temperatures, the foams with the addition of hydrotalcite (PU_10HT) showed higher conductivity than the reference foam (10 °C—0.02885 W m^–1^ K^–1^; 20 °C—0.03055 W m^–1^ K^–1^; 40 °C—0.03365 W m^–1^ K^–1^). In contrast, PU_10HS foam shows the highest values of all (10 °C—0.0336 W m^–1^ K^–1^; 20 °C—0.0352 W m^–1^ K^–1^; 40 °C—0.0388 W m^–1^ K^–1^). One of the reasons for such a phenomenon may be an inadequate foam structure, which could be indicated by a significantly reduced apparent density, in which there may be excessively large pores or open cells that create thermal bridges and allow heat to flow more freely between areas of the foam.

When analyzing the results of polyurethane foams with the addition of hazelnut shells and hydrotalcite, it can be observed that for all average measurement temperatures, all foams with a combination of fillers achieved higher thermal conductivity values compared to the reference foam. In this case, there is a trend of decreasing thermal conductivity as the hydrotalcite content increases and the hazelnut shell content decreases. This may be explained by the fact that the results of foams with hazelnut shell additives were significantly higher than the results of foams with mineral fillers, so foams with combinations of these additives have intermediate values. Similar relationships have also been observed in previous works [74,75]. For example, Paciorek-Sadowska et al. [76] showed that the thermal insulating properties of the obtained PU composites depend on the filler content. The addition of rapeseed cake particles in the amount of 60% wt.% increased the thermal conductivity of the obtained PU composites from 0.0341 to 0.0348 Wm^−1^ K^−1^.

Due to the widespread use of foams in a variety of industries, the evaluation of their combustion behavior can be essential in assessing the safety implications of such materials, as well as aiding in the implementation of fire prevention strategies. The polyurethane foams’ burning performance was investigated using a cone calorimeter, and the key parameters determined during the test were the ignition time (IT), total heat release (THR), total smoke release (TSR), carbon monoxide yield (COY), carbon dioxide yield (CO_2_Y), and maximum average rate of heat emission (MARHE). The results are shown in Table 6.

Based on the obtained data, it can be observed that the incorporation of mineral fillers affected the analyzed burning parameters. The addition of hydrotalcite resulted in an increase in ignition time, from 4 s for the reference foam to 8 s for the foams with added filler. The foams containing hydrotalcite exhibited lower values for THR and TSR parameters compared to the reference foam, which achieved values of 17.6 MJ m^–2^ and 492 m^2^ m^–2^, respectively, indicating that they emitted less heat and smoke during the combustion process.

The amount of carbon monoxide and carbon dioxide released was expressed by the parameters COY and CO_2_Y, and the ratio of these values provided information on the completeness of the combustion process. The foams with the addition of hydrotalcite exhibited reduced COY (0.24 kg kg^–1^) and CO_2_Y (2.61 kg kg^–1^) values compared to the reference foam (0.38 and 3.95 kg kg^–1^), as well as a slightly reduced COY/CO_2_Y ratio of 0.09 for PU_10HT, while it was 0.10 for PU_0.

The MARHE coefficient represents the highest average rate at which heat is released during combustion, and because it is used as a parameter for evaluating the intensity of a fire, materials are sought to achieve the lowest possible values [77]. Taking into account this parameter, it can be observed that the addition of mineral fillers reduces the MARHE values compared to the reference foam by 30% for PU_10HT.

When analyzing the burning behavior of polyurethane foams with the addition of hazelnut shells and mineral fillers, it can be observed that the ignition time increases from 4 s for the reference foam to 8 s for foams with the addition of hazelnut shells and hydrotalcite (PU_5HS_5HT and PU_2.5HS_7.5HT). These times are similar to the corresponding foams with 10 parts of mineral fillers.

In terms of THR and TSR parameters, all foams with the combinations of fillers showed lower values when compared to the reference foam (20.4 MJ m^–2^ and 750 m^2^ m^–2^), indicating lower heat and smoke emission during combustion. Foams with the addition of hazelnut shells and hydrotalcite achieved scores of 17.8, 17.2, and 17.3 MJ m^–2^, for PU_7.5HS_2.5HT, PU_5HS_5HT, and PU_2.5HS_7.5HT, respectively, similar to PU10HS foam reaching 17.9 MJ m^–2^ and PU_10HT foam reaching 17.6 MJ m^–2^. The highest value of the TSR parameter was achieved with PU_5HS_5HT foam (601 m^2^ m^–2^).

When analyzing the carbon monoxide yield, all foams with the hazelnut shell/mineral filler combinations showed lower results than PU_10HS (0.40 kg kg^–1^) and PU_0 (0.38 kg kg^–1^), and lower or comparable (for hazelnut shell/hydrotalcite foam) to the results of the corresponding foams with mineral fillers only, reaching between 0.24 and 0.35 kg kg^–1^. For carbon dioxide yield, all foams with the combinations of fillers performed lower than the reference foam (3.98 kg kg^–1^); in addition, hazelnut shell/hydrotalcite foams showed results of 2.90–3.10 kg kg^–1^, which were between PU_10HS foam (3.14 kg kg^–1^) and foams with hydrotalcite (2.61 kg kg^–1^) added individually.

Analyzing the MARHE results, it can be observed that all the foams with filler combinations obtained lower results than the reference foam (102 kW m^–2^); in addition, they obtained results between the extreme foams with 10 parts of single fillers, reaching results lower than PU_10HS (90 kW m^–2^) foam and higher than PU_10HT (71 kW m^–2^).

When analyzing the LOI results, it can be seen that all foams with filler combinations scored lower than the reference foam (19.8%). The PU_10HT foam showed the highest LOI value, indicating the best fire resistance (21.7%).

Based on the results of the burning behavior, it can be inferred that the foams with the addition of mineral fillers generally exhibited improved flammability parameters compared to those of the pure foam, except for the monoxide and dioxide yields for the foam with the addition of vermiculite. In general, mineral fillers can absorb a significant amount of heat, thereby lowering the temperature of the material itself and delaying ignition, and they can expand under heat and form a protective layer on the surface of the material, delaying degradation and ignition by slowing heat transfer and limiting oxygen access [78]. An additional factor may be the structure of the foams, as foams with added fillers had a higher density, and therefore a denser packing of material per unit volume and a lower oxygen content in the foam structure. Similar relationships have been demonstrated in previous work. For example, Kairyte et al. [79] showed that water-blown biopolyurethane (bioPUR) modified with lignin waste (LigW)/sodium silicate (LG) filler showed higher values of pHRR compared to the unmodified foam.

## 4. Conclusions

This article focused on the analysis of the properties of rigid polyurethane foams with the addition of combinations of hazelnut shells with hydrotalcite in various mutual weight ratios, including ratios of 1:3, 1:1, and 3:1 distributed over 10 parts by the weight of fillers contained in each foam. One of the main objectives of this part was to obtain innovative composites combining the use of natural additives (sustainable and environmentally conscious approach), fire safety (flame-retardant capabilities of mineral fillers), and maintaining optimal mechanical and performance properties. It was found that the addition of a combination of hazelnut shells and hydrotalcite resulted in an increase in the dynamic viscosity of the polyol systems, which affected the prolongation of the synthesis process compared to the reference foam. It also affected the cell structure of the filled foams, which exhibited smaller cell sizes and higher apparent density values. The mechanical properties were also influenced by the addition of the combinations of fillers and their effect on the cellular structure of the foams. In terms of hardness, all filled foams showed higher values than the reference foam and slightly worse or comparable performance to the reference foam for foams with higher hazelnut shell content and improved performance with increasing mineral filler content. In terms of water-related properties, the foams with the addition of a combination of fillers showed a relationship that as the density increased, the wetting angle values increased, and the water absorption decreased. Foams with added combinations of fillers generally exhibited improved thermal stability compared to the reference foam through higher temperatures of subsequent thermal decomposition stages and higher char residue content at 600 °C. In terms of burning behavior, foams containing hazelnut shell/hydrotalcite combinations exhibited improved flammability characteristics, extended ignition times, decreased total heat and total smoke release, and significantly lower values of the MARHE parameter, when compared to the pure foam. The results obtained indicated a longer time required for material ignition, and lower amounts of heat and smoke emitted during combustion, which may affect the spread of flames during a fire. For thermal conductivity, the great majority of foams with filler combinations had higher thermal conductivity than the reference foam.

## Figures and Tables

**Table 1:** Composition of polyurethane foams.

	Component [wt.%]								
	Polyol	Isocyanate	Surfactant	Catalysts		Foaming Agent		Nutshell	Mineral Filler
	Stapanpol PS-2352	Purocyn B	Tegostab B8513	Kosmos 75	Kosmos 33	Pentane	Water	Hazelnut Shells	Hydrotalcite
PU_0	100	160	2.5	6	0.8	11	0.5	0	0
PU_10HS	100	160	2.5	6	0.8	11	0.5	10	0
PU_7.5HS_2.5HT	100	160	2.5	6	0.8	11	0.5	7.5	2.5
PU_5HS_5HT	100	160	2.5	6	0.8	11	0.5	5	5
PU_2.5HS_7.5HT	100	160	2.5	6	0.8	11	0.5	2.5	7.5
PU_10HT	100	160	2.5	6	0.8	11	0.5	0	10

**Table 2:** Thermal stability results of walnut shells, vermiculite, and perlite.

Foam	1st Stage	2nd Stage	3rd Stage	Char Residue at 600 °C [wt.%]
	T_max_ [°C]			
HS	77 ± 2	347 ± 2	534 ± 3	5.7 ± 0.1
HT	235 ± 3	328 ± 3	426 ± 6	53.4 ± 0.1

**Table 3:** Characteristic times and maximum temperature of the synthesis of polyurethane foams with the addition of hazelnut shells and hydrotalcite.

	Start Time [s]	Expansion Time [s]	Stabilization Time [s]	Total Time [s]	Maximum Temperature [°C]	Dynamic Viscosity [mPa·s]
PU_0	29 ± 1	262 ± 7	351 ± 7	642 ± 7	121± 2	780 ± 12
PU_10HS	26 ± 1	285 ± 5	388 ± 6	699 ± 6	127 ± 1	2490 ± 15
PU_7.5HS_2.5HT	27 ± 1	285 ± 6	386 ± 6	698 ± 6	126 ± 2	2660 ± 10
PU_5HS_5HT	29 ± 2	282 ± 5	385 ± 5	696 ± 5	128 ± 1	2590 ± 13
PU_2.5HS_7.5HT	31 ± 1	276 ± 5	384 ± 5	691 ± 5	128 ± 2	2630 ± 15
PU_10HT	32 ± 2	268 ± 5	384 ± 6	684 ± 5	132 ± 1	2670 ± 15

**Table 4:** Structural properties of polyurethane foams with the addition of hazelnut shells and hydrotalcite.

	Cell Diameter [µm]	Anisotropy [-]	Apparent Density [kg·m^−3^]	Hardness [°Sh]
PU_0	510 ± 143	1.62 ± 0.14	35.49 ± 0.17	61.15
PU_10HS	528 ± 138	1.60 ± 0.16	32.51 ± 0.25	62.79
PU_7.5HS_2.5HT	485 ± 106	1.70 ± 0.23	33.17 ± 0.33	63.33
PU_5HS_5HT	474 ± 126	1.71 ± 0.52	34.44 ± 0.35	64.10
PU_2.5HS_7.5HT	470 ± 108	1.71 ± 0.26	35.91 ± 0.35	67.25
PU_10HT	439 ± 111	1.69 ± 0.19	40.45 ± 0.18	67.47

**Table 5:** Thermal stability results of polyurethane foams with the addition of hazelnut shells and mineral fillers.

Foam	1st Stage	2nd Stage	3rd Stage	Char Residue at 600 °C [wt. %]
	T_max_ [°C]			
PU_0	202 ± 2	310 ± 3	585 ± 5	22.3 ± 0.1
PU_10HS	207 ± 2	314 ± 2	599 ± 3	25.9 ± 0.1
PU_7.5HS_2.5HT	207 ± 2	314 ± 2	594 ± 4	24.0 ± 0.1
PU_5HS_5HT	221 ± 3	314 ± 3	599 ± 4	27.3 ± 0.1
PU_2.5HS_7.5HT	216 ± 3	314 ± 3	599 ± 3	30.1 ± 0.1
PU_10HT	212 ± 3	314 ± 3	604 ± 6	26.3 ± 0.1

**Table 6:** Burning behavior of polyurethane foams with the addition of hazelnut shells and hydrotalcite.

Foam	IT [s]	THR [MJ m^−2^]	TSR [m^2^ m^−2^]	COY [kg kg^−1^]	CO_2_Y [kg kg^−1^]	COY/CO_2_Y [-]	MARHE [kW m^−2^]	LOI [%]
PU_0	4 ± 0	20.4 ± 0.8	750 ± 8	0.38 ± 0.04	3.95 ± 0.03	0.10 ± 0.01	102 ± 4	19.8 ± 0.1
PU_10HS	6 ± 1	17.9 ± 0.6	364 ± 8	0.40 ± 0.03	3.14 ± 0.04	0.10 ± 0.01	90 ± 5	20.2 ± 0.1
PU_7.5HS_2.5HT	7 ± 1	17.8 ± 0.6	601 ± 9	0.35 ± 0.02	3.10 ± 0.04	0.08 ± 0.01	85 ± 5	20.8 ± 0.1
PU_5HS_5HT	10 ± 1	17.2 ± 0.7	593 ± 7	0.24 ± 0.02	3.12 ± 0.05	0.08 ± 0.01	83 ± 4	21.2 ± 0.1
PU_2.5HS_7.5HT	10 ± 1	17.3 ± 0.8	545 ± 6	0.25 ± 0.03	2.90 ± 0.04	0.09 ± 0.01	78 ± 4	21.2 ± 0.1
PU_10HT	8 ± 1	17.6 ± 0.8	492 ± 6	0.24 ± 0.04	2.61 ± 0.05	0.09 ± 0.01	71 ± 5	21.7 ± 0.1

## Data Availability

The original contributions presented in the study are included in the article, further inquiries can be directed to the corresponding author.
